# Effectiveness of lithium in subjects with treatment-resistant depression and suicide risk: a protocol for a randomised, independent, pragmatic, multicentre, parallel-group, superiority clinical trial

**DOI:** 10.1186/1471-244X-13-212

**Published:** 2013-08-13

**Authors:** Andrea Cipriani, Francesca Girlanda, Emilia Agrimi, Andrea Barichello, Rossella Beneduce, Irene Bighelli, Giulia Bisoffi, Alfredo Bisogno, Paola Bortolaso, Marianna Boso, Carmela Calandra, Liliana Cascone, Caterina Corbascio, Vincenzo Fricchione Parise, Francesco Gardellin, Daniele Gennaro, Batul Hanife, Camilla Lintas, Marina Lorusso, Chiara Luchetta, Claudio Lucii, Francesco Cernuto, Fiorella Tozzi, Alessandra Marsilio, Francesca Maio, Chiara Mattei, Daniele Moretti, Maria Grazia Appino, Michela Nosè, Guglielmo Occhionero, Duccio Papanti, Damiano Pecile, Marianna Purgato, Davide Prestia, Francesco Restaino, Tiziana Sciarma, Alessandra Ruberto, Stefania Strizzolo, Stefania Tamborini, Orlando Todarello, Simona Ziero, Spyridon Zotos, Corrado Barbui

**Affiliations:** 1 Dipartimento di Sanità Pubblica e Medicina di Comunità, Sezione di Psichiatria e Sezione di Psicologia Clinica, Università di Verona, Policlinico “G.B. Rossi” Piazzale L.A. Scuro, Verona, 10 – 37134, Italy; 2 Servizio Psichiatrico di Diagnosi e Cura, Istituti Ospitalieri di Cremona, Cremona, Italy; 3 IRCCS "Centro San Giovanni di Dio" FBF, Brescia, Italy; 4 Ufficio Supporto alla Ricerca e Biostatistica, Azienda Ospedaliera di Verona, Verona, Italy; 5 Dipartimento di Salute Mentale, UO Salute Mentale Cava de'Tirreni - Costa d'Amalfi, ASL Salerno, Italy; 6 Servizio Psichiatrico di Diagnosi e Cura Cittiglio, Psichiatria del presidio del Verbano, Ospedale di Circolo e Fondazione Macchi, Varese, Italy; 7 Dipartimento di Scienze Applicate e Psicocomportamentali, Sezione di Psichiatria, Università di Pavia e Centro Psico-Sociale di Pavia, Azienda Ospedaliera di Pavia, Pavia, Italy; 8 Azienda Ospedaliero Universitaria, "Policlinico-Vittorio Emanuele", Catania, Italy; 9 Dipartimento di Salute Mentale, Asl AT, Asti, Italy; 10 Asl Avellino (Regione Campania), U.O.C. di Salute Mentale di Avellino, Avellino, Italy; 11 Dipartimento di Salute Mentale, Ulss 6, Vicenza, Italy; 12 Azienda Ospedaliera SS Antonio e Biagio, Alessandria, Italy; 13 1° Servizio autonomo di Psichiatria, Ulss 20, Verona, Italy; 14 Università di Bari, Bari, Italy; 15 Dipartimento di Salute Mentale, Azienda per i Servizi Sanitari n°1 Triestina, Trieste, Regione FVG, Italy; 16 Azienda Usl 7, UFSMA Zona Altavaldelsa, Colle Val D’Elsa, Siena, Italy; 17 Dipartimento di Salute Mentale, Ulss 18, Rovigo, Italy; 18 Clinica Psichiatrica dell’Università di Genova, Genova, Italy; 19 Dipartimento di Salute Mentale, Centro di Salute Mentale di Finale Ligure, Asl n°2, Savona, Italy; 20 S.O.C Psichiatria Asti Centro-Nord, Asl AT, Asti, Italy; 21 Azienda Ospedaliera G. Salvini, U.O.P. n°62, Garbagnate Milanese, Italy; 22 Dipartimento di Medicina Clinica e Sperimentale, Sezione di Psichiatria, Psicologia Clinica e Riabilitazione Psichiatrica, Perugia, Italy; 23 Azienda Sanitaria Regionale del Molise, Servizio Psichiatrico di Diagnosi e Cura, Ospedale di Termoli, Termoli, Italy

**Keywords:** Randomised controlled trial, Lithium, Suicide, Deliberate self harm, Mortality, Depression, Treatment resistant, Antidepressant

## Abstract

**Background:**

Data on therapeutic interventions following deliberate self harm (DSH) in patients with treatment-resistant depression (TRD) are very scant and there is no unanimous consensus on the best pharmacological option for these patients. There is some evidence that lithium treatment might be effective in reducing the risk of completed suicide in adult patients with unipolar affective disorders, however no clear cut results have been found so far. The primary aim of the present study is to assess whether adding lithium to standard therapy is an effective treatment strategy to reduce the risk of suicidal behaviour in long term treatment of people with TRD and previous history of DSH.

**Methods/Design:**

We will carry out a randomised, parallel group, assessor-blinded superiority clinical trial. Adults with a diagnosis of major depression, an episode of DSH in the previous 12 months and inadequate response to at least two antidepressants given sequentially at an adequate dose for an adequate time for the current depressive episode will be allocated to add lithium to current therapy (intervention arm) or not (control arm). Following randomisation, treatment is to be taken daily for 1 year unless some clear reason to stop develops. Suicide completion and acts of DSH during the 12 months of follow-up will constitute the composite primary outcome. To preserve outcome assessor blindness, an independent adjudicating committee, blind to treatment allocation, will anonymously review all outcome events.

**Discussion:**

The results of this study should indicate whether lithium treatment is associated with lower risk of completed suicide and DSH in adult patients with treatment resistant unipolar depression, who recently attempted suicide.

**Trial registration:**

ClinicalTrials.gov identifier: NCT00927550

## Background

International guidelines usually define treatment-resistant depression (TRD) as an episode of major depression which fails to respond to two or more antidepressants given sequentially at an adequate dose and for an adequate time [1-3]. Interestingly, there is no unanimous consensus on the best pharmacological option in these patients: recommendations include switching to a different antidepressant, combining two antidepressants and adding another drug, like lithium, atypical antipsychotics, thyroid hormone, lamotrigine or pindolol [4]. Moreover, an important clinical feature frequently associated to such a difficult to treat population is that in TRD suicidal thoughts are common. They represent alarming psychopathological symptoms,[5] which may lead to deliberate self harm (DSH), suicide attempts and, in up to 10% of cases, to completed suicides [6].

Unfortunately, data on therapeutic interventions following non-fatal suicidal behaviour in patients with TRD are very scant. Hawton and colleagues carried out a Cochrane review to evaluate the effect of specific treatments for patients with depression and history of DSH [7,8]. Only three studies were found and the summary odds ratio (OR) indicated only a non-significant trend towards reduced repetition of DSH for antidepressant therapy compared with placebo (OR 0.83, 95% confidence interval (CI) 0.47 to 1.48). In addition to antidepressants, a possible beneficial effect in terms of suicidal behaviour has been suggested for lithium [9]. In a systematic review and meta-analysis of 32 trials recruiting patients with mood disorders, lithium was more effective than placebo on the risk of suicide, DSH and all-cause mortality, even though these differences were not statistically significant. Some limitations of this systematic review should be borne in mind: the included trials were not primarily designed to measure this outcome and heterogeneous patient populations were enrolled, including unipolar and bipolar patients. Another meta-analysis focused specifically on patients with recurrent, unipolar major depressive disorder and suggested for lithium an antisuicidal effect similar in magnitude to that found in bipolar disorders [10]. However, the major limitation of this analysis is that non-randomised studies were included in the analysis and this may have led to biased results.

In 2008, the results of a randomised, placebo-controlled trial focusing on the suicide preventive effects of lithium in patients with suicidal behaviour were published [11]. In this multi-centre trial (the SUPLI Study), patients with a recent history of a suicide attempt were treated double blind with lithium or placebo and followed up for 12 months. Of 167 patients included in the analysis, only 75% had a diagnosis of major depression: 20% of the whole sample had a diagnosis of adjustment disorder and 5% was suffering from dysthymia or related depressive disorders [12]. Survival analysis showed no significant difference of suicidal acts between lithium and placebo-treated individuals (adjusted hazard ratio 0.52; 95% CI 0.18 to 1.43). However, post hoc analysis revealed that all completed suicides had occurred in the placebo group accounting for a significant difference in incidence rates (p=0.049). Lauterbach and colleagues have suggested that lithium may specifically have suicide preventive effects because in their RCT all of the observed suicides occurred in the placebo group and none in the lithium group. However, the overall number of events was very small (all three suicides in the placebo group) and the lithium-treated individuals were at a higher risk as a result of the significant difference in number of previous suicide attempts which is the most reliable predictor for further suicidal behaviour [13]. Moreover, it also seems that the potential antisuicidal effect of lithium was independent from its mood-stabilising effects. Speculations about possible mechanisms of action involving the central serotonin system have been suggested, because lithium is thought to facilitate 5-HT neurotransmission [14] or may exert its antisuicidal effects influencing aggressive and impulsive traits that are linked to serotonergic dysfunction and also involved in the mediation of suicidal behaviour [15,16]. Evidence from this study suggested lithium treatment to be effective in reducing the risk of completed suicide in adult patients with unipolar affective disorders, however no clear cut results were found in terms of DSH (or suicidality) and the heterogeneity in terms of diagnosis of the recruited sample might have acted as an important confounder.

The primary aim of the present study is to assess whether adding lithium to standard therapy is an effective treatment strategy to reduce the risk of suicidal behaviour in long term treatment of people with TRD and previous history of DSH. Secondary aims of the study are: (a) to assess whether adding lithium has a specific impact in improving depressive symptoms; (b) to assess the long term tolerability profile of the lithium-augmentation strategy.

## Methods/Design

In a multi-centre, 12-month, superiority trial, we will randomise patients with TRD and history of DSH to (i) add lithium to usual treatment (add-on lithium strategy) or (ii) avoid lithium in usual treatment (without-lithium strategy). To be as close as possible to clinical practice, patients and clinicians will not be blind to treatments provided during the trial. However, to limit the potential bias introduced by lack of blindness, an independent adjudicating committee, blind to treatment allocation, will validate the events that will constitute the primary outcome. Patients will be assessed at baseline before randomisation and then every month after random allocation until the completion of the 12-month follow-up. All phases of the trial will be recorded following the CONSORT statement [17,18].

### Study population

Patients will be recruited in Italy. Community psychiatric services agreeing to take part to the study will be asked to recruit consecutive patients meeting the inclusion/exclusion criteria. Patients will be referred either from psychiatric wards or from community-based outpatient clinics. A total number of 50–60 psychiatric services will be involved. During the routine medical consultation relevant patients will be informed of and offered participation in the trial. If the patient agrees to participate in the trial, an appointment will be made for completion of relevant rating scales and questionnaires. Patients will be then randomly allocated to lithium plus usual treatment (either pharmacological or non-pharmacological) or to usual treatment alone.

To take part in the trial all patients must meet all of the inclusion criteria and none of the exclusion criteria, as follows:

#### Inclusion criteria

(a) Diagnosis of major depression (clinical diagnosis, guided by DSM-IV criteria).

(b) An episode of DSH in the previous 12 months.

(c) Inadequate response to at least two antidepressants given sequentially at an adequate dose for an adequate time for the current depressive episode.

(d) Uncertainty about which treatment arm would be best for the participant.

(e) Age 18 or above.

(f) To sign written informed consent.

#### Exclusion criteria

(a) A primary diagnosis of any concurrent Axis I disorder (according to DSM-IV criteria) other than major depression (by contrast, any concurrent DSM-IV Axis II disorder will not constitute an exclusion criterion).

(b) Previous exposure to lithium associated with lack of efficacy or adverse reactions.

(c) Clinical conditions contraindicating lithium (i.e., thyroid or kidney disease/abnormalities).

(d) Pregnant/lactating women and women of childbearing potential not practicing a reliable method of contraception.

### Interventions

Lithium is currently marketed in Italy for the treatment of major depression and it will be prescribed according to Italian usual care. Patients allocated to lithium will be administered an oral starting dose ranging between 150 and 300 milligrams (according to clinical judgement). Suggested final oral dose will have to achieve plasma levels of 0.4 to 1.0 mmol/L. Clinicians will be free of increasing or decreasing the dose according to clinical status and circumstances. Dose changes will be recorded. Following randomisation, treatment is to be taken daily for 1 year unless some clear reason to stop develops. Patients allocated to the lithium arm will receive usual pharmacological and non-pharmacological treatment as clinically indicated. Any other pharmacological treatment will be allowed.

Patients allocated to the control arm will receive usual pharmacological and non-pharmacological treatment as clinically indicated. Patients allocated to the control arm will not be allowed to receive lithium. Any other pharmacological treatment will be allowed.

Routine care outside the trial continues as usual. During the study, participants will be seen as often as clinically indicated with no extra visits required for the trial.

### Baseline and follow up assessments

Before entering the study, patients will be asked to provide informed consent to participate. The following information will be collected at baseline: sociodemographic and clinical characteristics, diagnosis according to the Mini Neuropsychiatry Interview (MINI) [19], laboratory and electrocardiography (ECG) parameters, severity of illness according to the Quick Inventory Depression Scale (QIDS) [20], a self-rated instruments that has been shown to have good psychometric properties [21]. Detailed information will be gathered on previous DSH acts, as well.

Follow-up data will be obtained each month after random allocation using an electronic form, as follows: any death or DSH, lithium oral dose and plasma level (if applicable), concomitant drug treatments, QIDS scores, adverse events and information on laboratory and ECG parameters.

### Random allocation procedure

Patients will be randomly assigned to one of the two treatment groups with an equal probability of assignment to each treatment (allocation ratio 1:1). A centralised randomisation procedure will be employed. The trial biostatistician will prepare the sequence of treatments randomly permuted in blocks of unequal size. The allocation will be stratified by presence or absence of Axis II diagnosis *(patients with unipolar major depression AND personality disorder vs patients with unipolar major depression WITHOUT personality disorder)* because this variable may have an impact on the study outcomes (see Discussion).The randomisation schedule will be generated using nQuery Advisor, release 7.0. Recruiting physicians will contact an administrator at the World Health Organisation Collaborative Centre for Research and Training in Mental Health and Service Evaluation of the University of Verona who will access a computerised system that will provide, after information on the enrolled participant is entered and inclusion criteria are verified, the patient's identification number and the allocated treatment.

### Outcomes

Suicide completion and acts of DSH during the 12 months of follow-up will constitute the composite primary outcome. The term “suicide” is defined as an act with a fatal outcome, deliberately initiated and performed by the person with the knowledge or expectation of its fatal outcome. DSH is defined as intentional self-poisoning or self-injury, irrespective of motivation, as the intention to end life may be absent or present to a variable degree [7,8]. To preserve outcome assessor blindness, an independent adjudicating committee (three clinical psychiatrists, with no links to the study and blind to treatment allocation) will anonymously review all deaths and all hazardous acts reported during the 12 months of follow-up by the treating clinicians. The committee will have the role of identifying, among deaths, the cases of suicide, and among hazardous acts those that constitute acts of DSH. Only the cases of suicide and those of DSH validated by the committee will constitute the primary outcome.

As secondary outcomes, we will consider number of events (acts of DSH), number of suicides and all-cause mortality, change in severity of depressive symptoms from baseline to endpoint and adverse reactions during the 12 months of follow-up.

### Power analysis and sample size calculation

When we first drafted the study protocol, the sample size calculation was based on data from three antidepressant clinical trials that employed completed and attempted suicide as the primary endpoint [7,8] (for details, see clinicaltrials.gov, ClinicalTrials.gov identifier: NCT00927550). However, the most recently published evidence has shown that the event rate of completed and attempted suicide in patients with unipolar, recurrent depression (with or without Axis II diagnosis) is higher than expected and can be more than 35% at 12 months [22,23]. According to this data, hypothesising that lithium augmentation (experimental group) would show a clinically significant advantage by producing an event rate of 10% [7,8], a sample size of 210 patients (105 in each group) achieves 80% power for a 0.05 level of two-sided log-rank test (31 events expected).

### Statistical analysis

The statistical analysis will be masked, i.e. the trial biostatistician will be blinded to the treatment groups until the analysis has been completed. All analyses will be performed using Stata/SE, Release 11.1. Data lock will occur at 12 months, when data will be available for all participants. The pattern of missing values will be explored and interactions between treatment group and non-completers/completers will be examined with respect to demographic variables and baseline disease assessment.

The intention-to-treat (ITT) population, consisting of all subject randomly assigned to the competitive treatment strategies, will be used for the analysis of primary and secondary outcomes. Patients with missing values and patients lost during follow-up will contribute to the analysis of the primary and secondary outcomes only for the time during which data are available (censoring). Missing values in depressive symptom ratings will be imputed using the last observation carried forward (LOCF) approach: depressive ratings will be carried forward from the last available assessment to the 12-month follow up assessment. Additionally, patients in each arm will be always analysed according to the corresponding treatment group’s allocation at baseline.

In order to check the consistency of this ITT approach, the primary outcome will then be analysed using a per-protocol (PP) approach. The analysis of the PP population will be used for confirmatory purposes only. If less than 5% of patients switch from the allocated treatment to the competitive treatment, the PP analysis will not be performed.

Kaplan-Meier estimates for the time from randomised treatment assignment until the first event that constitutes the primary outcome will be plotted to compare the treatment’s effect, and log-rank test will be performed to test for differences. A Cox proportional hazard model will be used to explore the effect of possible confounding or interaction factors. Gender and age will be included into the model regardless of statistical significance criteria. The proportional hazard assumption of the effects will be tested. No interim analysis is planned for this trial.

Time from randomised treatment assignment until all-cause mortality during the 12 months of follow-up will be analysed through a log-rank test for difference in survival between the two groups. The same method will be applied for the following secondary outcomes: suicide mortality, deliberate self-harm. Multivariate analyses will be performed to explore the possible role of prognostic factors on outcomes.

Change in severity of depressive symptoms from baseline to 12 months will be analysed as a continuous variable and as a dichotomous variables, where individuals showing an improvement of al least 50% will be considered treatment responders. Change in severity of illness at 12-months will be compared between the two groups of treatment through appropriate statistical methods for repeated measurements (paired t-test or McNemar non parametric test according to the variables distribution). The number of events/outcomes of interest (suicide or DSH) will be compared between the two groups of treatment through the chi-square test (or the Fisher’s exact test when appropriate). When possible, a multivariate analysis will be performed through a Poisson regression model with a robust error variance.

The proportion of patients with adverse reactions during the study will be compared between the two groups of treatment through the chi-square test (or the Fisher’s exact test when appropriate).

#### Data management

All study data will be entered in a computerised database and stored by the World Health Organisation Collaborative Centre for Research and Training in Mental Health and Service Evaluation of the University of Verona. The correctness and consistency of the data will be ensured by the double-entry technique and by a set of electronic and manual edit checks. In accordance with the Declaration of Helsinki, patients’ confidentiality will be preserved at all times and the contents of the recruitment and follow-up forms will not be disclosed to any third party. The data collected in the study corresponding to a patient will be recorded anonymously.

### Ethical aspects

This study will be conducted according to globally accepted standards of good clinical practice (as defined in the ICH E6 Guideline for Good Clinical Practice, 1 May 1996), in agreement with the Declaration of Helsinki and in keeping with local regulations. Study investigators will ensure that all persons assisting with the trial are adequately qualified, informed about the protocol, any amendments to the protocol, the study treatments, and their trial-related duties and functions. The coordinating centre will maintain a list of sub-investigators and other appropriately qualified persons involved in the study. Before being enrolled in the study, subjects will consent to participate after the nature, scope, and possible consequences of the clinical trial have been explained in a form understandable to them. An informed consent document that includes both information about the study and the consent form will be given to participants. This document will contain all the elements required by the ICH E6 Guideline for Good Clinical Practice and any additional elements required by local regulations. The document will be in a language understandable to the participants and will specify who informed the subject. The person who informs the subject will be a physician. After reading the informed consent document, the subject, or his/her legal representative, must give consent in writing. The subject’s consent must be confirmed at the time of consent by the personally dated signature of the subject and by the personally dated signature of the person conducting the informed consent discussion.

### Ethical approval document

The study has been approved by the Ethics Committee in Verona (coordinating centre) on May 6^th^ 2009 (EUDRACT number 2009-011409-17.

According to the ICH E6 Guideline for Good Clinical Practice, subjects who will be enrolled in the trial with the consent of the subjects’ legally acceptable representative will be informed about the trial to the extent compatible with the subjects’ understanding and, if capable, the subject will be asked to sign and personally date the written informed consent.

## Discussion

The results of this study should indicate whether lithium treatment is associated with lower risk of completed suicide and DSH in adult patients with treatment resistant unipolar depression, who recently attempted suicide. At present, evidence is lacking to indicate the most effective forms of pharmacological treatment for depressed patients with recent history of DSH. To answer this question is an important clinical issue, given the size of the DSH population and the risks of subsequent self-harm, including suicide. To our knowledge, this is the first study that will prospectively identify suicidal behaviour as an outcome measure and systematically assess it in a randomised design, recruiting a well-characterised and homogeneous sample of patients with treatment resistant major depression, with a high risk for suicidal behaviour. Moreover, as most meta-analyses of randomised clinical trials have not detected a reduction in number of suicides or suicide attempts in studies of antidepressants or anticonvulsants for mood disorders [24], the prospect of an agent with suicide-preventive properties is bound to have an enormous impact on the development of treatment strategies and clinical guidelines [25].

Designing our study, we aimed at being as close as possible to the real-world clinical setting. The recruiting centres will all be community based psychiatric services, where patients are not selected by diagnosis and are a representative subgroup of the general population. Even though this may enhance the external validity of the study and the generalisability of its findings, the heterogeneity of patient population might be an issue. High rate of DSH is usually found in patients with personality disorders (most of all, borderline disorder). This has clinical implications because patients with Axis II disorder comorbidity are clinically different from patients with major depression alone and borderline personality disorder can confer additional risk for suicidal ideation and DSH [26]. For this reason we stratified the randomisation, in order to have an equal distribution of treatment regimens in the two subgroups.

We are aware that to have standard care as comparison intervention may cause additional heterogeneity, as standard care may probably vary from centre to centre. Variability in standard aftercare of DSH patients in different regions (even though belonging to the same country, that is, Italy) may influence the relative effectiveness of experimental interventions in particular settings. In the final publication we will report all details of the standard care, as it is provided by clinicians who will prospectively collect precise data about the nature of the treatments patients will receive.

## Competing interests

The authors declare that they have no competing interests.

## Authors’ contributions

All authors have made substantial contributions to conception and design, or acquisition of data. AC, CB, FG and GB have been involved in drafting the manuscript and all remaining authors have it critically for important intellectual content. All authors read and approved the final manuscript.

## Pre-publication history

The pre-publication history for this paper can be accessed here:

http://www.biomedcentral.com/1471-244X/13/212/prepub

## References

[B1] Nierenberg AA Amsterdam JD Treatment-resistant depression: definition and treatment approaches J Clin Psychiatry 1990 51 Suppl 39 47 2112132

[B2] Bauer M Whybrow PC Angst J Versiani M Moller HJ World federation of societies of biological psychiatry (WFSBP) guidelines for biological treatment of unipolar depressive disorders, part 1: acute and continuation treatment of major depressive disorder World J Biol Psychiatry 2002 3 5 43 10.3109/15622970209150599 12479086

[B3] Carvalho AF Cavalcante JL Castelo MS Lima MC Augmentation strategies for treatment-resistant depression: a literature review J Clin Pharm Ther 2007 32 415 428 10.1111/j.1365-2710.2007.00846.x 17875106

[B4] Papakostas GI Fava M Thase ME Treatment of SSRI-resistant depression: a meta-analysis comparing within- versus across-class switches Biol Psychiatry 2008 63 699 704 10.1016/j.biopsych.2007.08.010 17919460

[B5] Fava M Diagnosis and definition of treatment-resistant depression Biol Psychiatry 2003 53 649 659 10.1016/S0006-3223(03)00231-2 12706951

[B6] Cipriani A Barbui C Geddes JR Suicide, depression, and antidepressants BMJ 2005 330 373 374 10.1136/bmj.330.7488.373 15718515 PMC549094

[B7] Hawton K Arensman E Townsend E Bremner S Feldman E Goldney R Deliberate self harm: systematic review of efficacy of psychosocial and pharmacological treatments in preventing repetition BMJ 1998 317 441 447 10.1136/bmj.317.7156.441 9703526 PMC28637

[B8] Hawton K Townsend E Arensman E Gunnell D Hazell P House A Psychosocial versus pharmacological treatments for deliberate self harm Cochrane Database Syst Rev 2000 CD001764 10796818 10.1002/14651858.CD001764

[B9] Cipriani A Pretty H Hawton K Geddes JR Lithium in the prevention of suicidal behavior and all-cause mortality in patients with mood disorders: a systematic review of randomized trials Am J Psychiatry 2005 162 1805 1819 10.1176/appi.ajp.162.10.1805 16199826

[B10] Guzzetta F Tondo L Centorrino F Baldessarini RJ Lithium treatment reduces suicide risk in recurrent major depressive disorder J Clin Psychiatry 2007 68 380 383 10.4088/JCP.v68n0304 17388706

[B11] Lauterbach E Ahrens B Felber W Oerlinghausen BM Kilb B Bischof G Suicide prevention by lithium SUPLI–challenges of a multi-center prospective study Arch Suicide Res 2005 9 27 34 10.1080/13811110590512886 16040577

[B12] Lauterbach E Felber W Muller-Oerlinghausen B Ahrens B Bronisch T Meyer T Adjunctive lithium treatment in the prevention of suicidal behaviour in depressive disorders: a randomised, placebo-controlled, 1-year trial Acta Psychiatr Scand 2008 118 469 479 10.1111/j.1600-0447.2008.01266.x 18808400

[B13] Brown GK Beck AT Steer RA Grisham JR Risk factors for suicide in psychiatric outpatients: a 20-year prospective study J Consult Clin Psychol 2000 68 371 377 10883553

[B14] Young LT Warsh JJ Kish SJ Shannak K Hornykeiwicz O Reduced brain 5-HT and elevated NE turnover and metabolites in bipolar affective disorder Biol Psychiatry 1994 35 121 127 10.1016/0006-3223(94)91201-7 7513191

[B15] Mann JJ Waternaux C Haas GL Malone KM Toward a clinical model of suicidal behavior in psychiatric patients Am J Psychiatry 1999 156 181 189 9989552 10.1176/ajp.156.2.181

[B16] Corruble E Hatem N Damy C Defense styles, impulsivity and suicide attempts in major depression Psychopathology 2003 36 279 284 10.1159/000075185 14646450

[B17] Moher D Schulz KF Altman DG The CONSORT statement: revised recommendations for improving the quality of reports of parallel-group randomised trials Lancet 2001 357 1191 1194 10.1016/S0140-6736(00)04337-3 11323066

[B18] Zwarenstein M Treweek S Gagnier JJ Altman DG Tunis S Haynes B Improving the reporting of pragmatic trials: an extension of the CONSORT statement BMJ 2008 337 a2390 10.1136/bmj.a2390 19001484 PMC3266844

[B19] Sheehan DV Lecrubier Y Sheehan KH Amorim P Janavs J Weiller E The mini-international neuropsychiatric interview (M.I.N.I.): the development and validation of a structured diagnostic psychiatric interview for DSM-IV and ICD-10 J Clin Psychiatry 1998 59 Suppl 20 22 33 9881538

[B20] Rush AJ Trivedi MH Ibrahim HM Carmody TJ Arnow B Klein DN The 16-item quick inventory of depressive symptomatology (QIDS), clinician rating (QIDS-C), and self-report (QIDS-SR): a psychometric evaluation in patients with chronic major depression Biol Psychiatry 2003 54 573 583 10.1016/S0006-3223(02)01866-8 12946886

[B21] Rush AJ Trivedi MH Wisniewski SR Stewart JW Nierenberg AA Thase ME Bupropion-SR, sertraline, or venlafaxine-XR after failure of SSRIs for depression N Engl J Med 2006 354 1231 1242 10.1056/NEJMoa052963 16554525

[B22] Nitwoski D Petermann F Non-suicidal self-injury and comorbid mental disorders: a review Fortschr Neurol Psychiat 2011 79 9 20 10.1055/s-0029-1245772 21104583

[B23] de Klerk S van Noorden MS van Giezen AE Spinhoven P den Hollander-Gijsman ME Giltay EJ Speckens AE Zitman FG Prevalence and correlates of lifetime deliberate self-harm and suicidal ideation in naturalistic outpatients: the leiden routine outcome monitoring study J Affect Disord 2011 133 1–2 257 264 21463900 10.1016/j.jad.2011.03.021

[B24] Mann JJ Apter A Bertolote J Suicide prevention strategies: a systematic review JAMA 2005 294 2064 2074 10.1001/jama.294.16.2064 16249421

[B25] Muller-Oerlinghausen B Berghofer A Ahrens B The antisuicidal and mortality-reducing effect of lithium prophylaxis: consequences for guidelines in clinical psychiatry Can J Psychiatry 2003 48 433 439 12971012 10.1177/070674370304800702

[B26] Sharp C Green KL Yaroslavsky I Venta A Zanarini MC Pettit J The incremental validity of borderline personality disorder relative to major depressive disorder for suicidal ideation and deliberate self-harm in adolescents J Pers Disord 2012 26 6 927 938 10.1521/pedi.2012.26.6.927 23281677 PMC3752939

